# Implantable Drainage After Major Abdominal Surgery in Compromised Patients

**DOI:** 10.1155/1990/98437

**Published:** 1990

**Authors:** Roland Andersson, Bengt Jeppsson, Anna Holmberg, Stig Bengmark

**Affiliations:** 1 Department of Surgery Lund University Sweden; 2 Department of Surgery Lund University Lund S-221 85 Sweden

## Abstract

The risk of superinfection following routine abdominal drainage after major surgery is debated. Especially in patients with malignant diseases and a compromised host defense, this might be a factor increasing morbidity and mortality. During a 3-year period (1986–1988) 41 patients operated on for malignant abdominal conditions received a peritoneal catheter connected to a subcutaneous portal inserted in order to participate in a trial on postoperative intraperitoneal chemotherapy using 5- Fluorouracil. No abdominal drains were inserted. In 15 patients, the subcutaneous portal was used for evacuation of postoperative fluid accumulation within the abdomen. The mean age was 53 (range 41–70) years. Inserted catheters were used for drainage up to 14 days postoperatively. The daily amount of fluid drained varied from 20 to 2 000 ml with a mean of 610 ml/patient and day. One patient required removal of the catheter due to infection around the subcutaneous chamber. Otherwise, the catheter system was not associated with any other complications or complaints. One patient developed a postoperative left subphrenic abscess drained percutaneously by the guidance of ultrasonography, a complication that could not be attributed to the catheter system but merely to the major operation *per se*. An implantable device for peritoneal access thus also seem useful for drainage of postoperative fluid collection, as evaluated in this preliminary report.


*HPB Surgery*, 1990, Vol. 2, pp. 261–264
Reprints available directly from the publisher
Photocopying permitted by license only

© 1990 Harwood Academic Publishers GmbH
Printed in the United Kingdom

### IMPLANTABLE DRAINAGE AFTER MAJOR ABDOMINAL SURGERY IN COMPROMISED PATIENTS

ROLAND ANDERSSON, BENGT JEPPSSON, ANNA HOLMBERG and STIG BENGMARK
*Department of Surgery, Lund University, Sweden*

*(Received 9 January 1990)*

The risk of superinfection following routine abdominal drainage after major surgery is debated. Especially in patients with malignant diseases and a compromised host defense, this might be a factor increasing morbidity and mortality. During a 3-year period (1986–1988) 41 patients operated on for malignant abdominal conditions received a peritoneal catheter connected to a subcutaneous portal inserted in order to participate in a trial on postoperative intraperitoneal chemotherapy using 5-Fluorouracil. No abdominal drains were inserted. In 15 patients, the subcutaneous portal was used for evacuation of postoperative fluid accumulation within the abdomen. The mean age was 53 (range 41–70) years. Inserted catheters were used for drainage up to 14 days postoperatively. The daily amount of fluid drained varied from 20 to 2 000 ml with a mean of 610 ml/patient and day. One patient required removal of the catheter due to infection around the subcutaneous chamber. Otherwise, the catheter system was not associated with any other complications or complaints. One patient developed a postoperative left subphrenic abscess drained percutaneously by the guidance of ultrasonography, a complication that could not be attributed to the catheter system but merely to the major operation *per se*. An implantable device for peritoneal access thus also seem useful for drainage of postoperative fluid collection, as evaluated in this preliminary report.

KEY WORDS: Major abdominal surgery, compromised patients, implantable drainage.

### INTRODUCTION

Prophylactic drainage following major abdominal surgery has previously been routinely used. The benefit of routine drainage following major abdominal surgery has, however, been questioned and drainage as a port of entry for bacteria has been proposed[1]. In patients with malignant diseases and a compromised host defense against infection, a percutaneous drain carries the risk of introducing bacteria intraabdominally and thereby increasing morbidity and mortality.

Additional methods of treating these abdominal malignancies are sought for. Intraperitoneal chemotherapy through an implantable system for peritoneal access has been under trial during recent years. Probably the most experience hitherto concerns the use of intraperitoneal chemotherapy for ovarian carcinoma,[2–5] though also trials on the value of intraperitoneal 5-Fluorouracil in patients with colorectal liver metastases[6–8] have been performed. The system used has mainly been a

Correspondence to: Roland Andersson, M.D., Department of Surgery, Lund University, S-221 85 LUND, Sweden.

permant peritoneal dialysis (Tenckhoff) catheter placed intraperitoneally connected with a totally implanted system placed subcutaneously. In the present study, patients receiving this kind of system for peritoneal access in order to participate in a postoperative trial on intraperitoneal chemotherapy, had no other abdominal drains inserted following surgery and thus the implantable peritoneal access system was also used for postoperative drainage.

### PATIENTS AND METHODS

During the period 1986–1988, a total of 41 patients (27 men, 14 women) had peritoneal catheters connected with a subcutaneous portal (Trava-Port, Kabi-Baxter, Stockholm, Sweden) inserted in addition to surgery, as the patients were going to participate in a trial of postoperative intraperitoneal chemotherapy (using 5-Fluorouracil). Out of these, 15 patients (8 men, 7 women) had the implantable system for peritoneal access used for drainage of postoperative fluid accumulations, as no other abdominal drains were used. The mean age in this group was 57 (range 41–70) years. The surgical procedures performed were liver resection (n = 6), pancreatectomy (n = 4) or preparation for temporary liver dearterialization with application of a vascular occluding device around the hepatic artery (n = 5). In the remaining 26 patients, 8 had an additional "conventional" abdominal drainage (Penrose$^R$) inserted, while in the other 18 patients the implantable device was not used for drainage of abdominal fluid as the peroperative surgical trauma was less extensive and the postoperative course was uneventful, not necessitating the need for drainage. The percutaneous injection portal, consisting of a conical, stainless steel chamber with a self-sealing silicone rubber septum connected with a silicone catheter was placed laterally to the left or right border of the rectus abdominis muscle at, or slightly above, the level of the umbilicus, and brought through a subcutaneous channel before entering the abdominal cavity. The system is simply filled with saline but is otherwise without maintenance requirements.

### RESULTS

Fifteen out of the total of 41 patients (37%) had the peritoneal access system used for the evacuation of postoperative fluid accumulations. In these cases, the inserted catheters were used for drainage up to 14 days postoperatively. The mean daily amount of drained fluid per patients was 610 ml with a range from 20 to 2 000 ml. The fluid that was drained was in general clear and uninfected. The subcutaneous portal and catheter system seemed in general to be well tolerated and complications were few. One patient had the catheter removed due to a local infection around the subcutaneous port. One patient developed a subphrenic abscess drained percutaneously with the guidance of ultrasonography, a complication that was not contributed to by the catheter system *per se*, but merely to the major operation performed.

Among the remaining 26 patients, 3 abdominal abscesses were diagnosed in the postoperative period, 2 managed percutaneously and 1 surgically. No correlation between the use of conventional drainage and abscess formation could be seen in this small series.

### DISCUSSION

Intraperitoneal administration of cytostatic agents, for example, using the described system is simple, without a need for an indwelling arterial catheter and infusion pump. The system provides a more uniform delivery to the hepatic parenchyma and it achieves the highest plasma levels in the portal system. It can also be used for treating concomitant intraabdominal disease, which may benefit from intraperitoneal therapy. The exact indications for using this route of administering cytostatic agents, however, remain to be defined.

Implantable ports allow flow rates up to 37 ml/minute with 1 meter of gravity pressure[9]. This permits instillation of 2 litres of fluid in approximately 60 minutes and drainage of the same volume in 60–90 minutes. Furthermore, evacuation can be enhanced if the needle is attached to a vacuum system. By flushing the catheter, plugging of the lumen with fibrinous debris does not seem to be a problem and as the need for evacuation of fluid accumulation in the postoperative period is fairly limited in time, i.e. drainage is generally needed only for the first postoperative days, the walling off of the catheter within the peritoneal cavity does not seem to constitute a major problem. Furthermore, before initiating intraperitoneal drug administration at our institution, an abdominal scintigraphy using intraperitoneal $Tc^{99m}$ sulphur colloid is performed in order to guarantee a normal intraabdominal spread.

By using the implanted system for peritoneal access, there is also the possibility for postoperative evacuation of fluid from within the abdominal cavity following major surgery, as is shown in the present study. Thereby, the risk of superinfection by introducing bacteria through an open abdominal drainage system in these compromised patients is diminished. Maybe any drainage of abdominal fluid at all could have been omitted in a few patients in the present series, but drainage volumes were in general large, probably necessitating some kind of abdominal drainage in the early postoperative period. Further on, the mobilization of patients is facilitated by the absence of external drainage, thereby hopefully decreasing the incidence of postoperative complications and reducing the length of the hospital stay.

### *References*

1. Cerise, E.J., Pierce, W.A. *et al.* (1970) Abdominal drains; Their role as a source of infection following splenectomy. *Ann. Surg.* **171**, 764–769
2. Markman, M., Howell, S.B., Lucas, W.E., Pfeifle, C.E., Green, M.R. (1984) Combination intraperitoneal chemotherapy with cisplatin, cytarabine and doxorubicin for refractory ovarian carcinoma and other malignancies principally confined to the peritoneal cavity. *J. Clin. Oncol*, **2**, 1321–1326
3. Ten Bokkel Huinink, W.W., Dubbelman, R., Aartsen, E., Franklin, H., McVie, J.G. (1985) Experimental and clinical results with intraperitoneal cisplatin. *Semin. Oncol.* suppl. **4**, XII (3), 43–46
4. Abrams, J.S. (1986) Current status of intraperitoneal chemotherapy in ovarian cancer. *Louvain. Med.*, **105**, 159–165
5. Ozols, R. (1985) Intraperitoneal chemotherapy in the management of ovarian cancer. *Semin. Oncol.* suppl. **4**, XIII (3) 75–80
6. Sugarbaker, P.H., Gianola, F.J., Speyer, J.L., Wesley, R., Barofsky, I., Myers, C.E. (1985) Prospective randomized trial of intravenous vs intraperitoneal 5-FU in patients with advanced primary colon or rectal cancer. *Surgery*, **98**, 414–422

7. August, D.A., Sugarbaker, P.H., Ottow, R.T., Gianola, F.J., Schneider, P.D. (1985) Hepatic resection of colorectal metastases: influence of clinical factors and adjuvant intraperitoneal 5-Fluorouracil via Tenckhoff catheter on survival. *Ann. Surg.* **201**, 210–218
8. Ekberg, H., Tranberg, K.-G., Persson, B., *et al.* (1988) Intraperitoneal infusion of 5-FU in liver metastases from colorectal cancer. *J. Surg. Oncol.* **37**, 94–99
9. Pfeifle, C.E., Howell, S.B., Markman, M., Lucas, W.E. (1984) Totally implantable system for peritoneal access. *J. Clin. Oncol.* **2**, 1277–1280

